# OP09 Enhancing Generic Medicine Pricing Frameworks: Sustainable Solutions For Low- And Middle-Income Countries

**DOI:** 10.1017/S0266462325100688

**Published:** 2025-12-29

**Authors:** Birol Tibet

## Abstract

**Introduction:**

Generic medicines form the backbone of affordable health care in low- and middle-income countries (LMICs), constituting a significant portion of pharmaceutical consumption. However, issues such as fragmented pricing policies and insufficient regulatory frameworks hinder equitable access and affordability. This study evaluated pricing models that address these challenges by integrating best practices, including tiered pricing mechanisms and enhanced transparency. The aim was to guide policymakers toward sustainable solutions that improve medicine affordability, equity, and market resilience.

**Methods:**

A comparative analysis was conducted across 20 countries, chosen for their diverse healthcare profiles and potential to be best practice examples for LMIC contexts. Key elements analyzed included pricing mechanisms (e.g., tiered pricing, external reference pricing, internal reference pricing), regulatory frameworks, and incentives for local production. Data were gathered from government reports, healthcare provider feedback, and industry insights. A policy evaluation framework was applied to assess each model’s effectiveness in addressing affordability, accessibility, and equity. The analysis prioritized generalizability to LMICs while accounting for contextual adaptations such as regional economic challenges and varying levels of regulatory capacity.

Supported by TUBITAK 2224-A Program for participation in HTAi 2025; it had no role in the study conduct.

**Results:**

The findings revealed that tiered pricing structures significantly enhanced affordability by incentivizing market competition and reducing prices as generics enter. Transparent regulatory practices, including centralized databases and consistent price reviews, mitigated regional price disparities and increased trust among stakeholders. Countries fostering domestic production reduced import dependencies, stabilizing supply chains and ensuring medicine availability. These strategies, when applied collectively, have great potential to improve equitable access to generics in resource-constrained settings. The results highlighted the importance of adapting global best practices to the economic and healthcare realities of LMICs, emphasizing sustainability and resilience in pharmaceutical markets.

**Conclusions:**

Integrating tiered pricing frameworks, regulatory transparency, and local production incentives provided a pragmatic pathway to improve generic medicine access in LMICs. These approaches align with global best practices and address affordability and equity challenges. Policymakers should prioritize such strategies while leveraging stakeholder collaboration. Future research should examine dynamic pricing models and their long-term impacts on market stability and healthcare outcomes.

